# Rising Incidence of *Sporothrix brasiliensis* Infections, Curitiba, Brazil, 2011–2022

**DOI:** 10.3201/eid2907.230155

**Published:** 2023-07

**Authors:** Regielly C.R. Cognialli, Diego H. Cáceres, Fernanda de A.G.D. Bastos, Francelise B. Cavassin, Bruno P.R. Lustosa, Vânia A. Vicente, Giovanni L. Breda, Izabella Santos-Weiss, Flávio Queiroz-Telles

**Affiliations:** Federal University of Paraná Program in Internal Medicine and Health Science, Curitiba, Brazil (R.C.R. Cognialli, F. de A.G.D. Bastos);; Federal University of Paraná Hospital de Clínicas, Curitiba (R.C.R. Cognialli, G.L. Breda);; Center of Expertise in Mycology Radboudumc/CWZ, Nijmegen, the Netherlands (D.H. Cáceres);; Universidad del Rosario Studies in Translational Microbiology and Faculty of Medical Sciences, Emerging Diseases Research Group, Bogota, Colombia (D.H. Cáceres);; Faculdades Pequeno Príncipe, Curitiba (F.B. Cavassin);; Federal University of Paraná Basic Pathology Department, Curitiba (B.P.R. Lustosa, V.A. Vicente);; Federal University of Paraná Department of Clinical Analysis, Curitiba (I. Santos-Weiss);; Federal University of Paraná Department of Public Health, Curitiba (F Queiroz-Telles)

**Keywords:** sporotrichosis, zoonoses, mycoses, Sporothrix brasiliensis, cat-transmitted sporotrichosis, fungi, Brazil

## Abstract

Zoonotic outbreaks of sporotrichosis are increasing in Brazil. We examined and described the emergence of cat-transmitted sporotrichosis (CTS) caused by the fungal pathogen *Sporothrix brasiliensis*. We calculated incidence and mapped geographic distribution of cases in Curitiba, Brazil, by reviewing medical records from 216 sporotrichosis cases diagnosed during 2011–May 2022. Proven sporotrichosis was established in 84 (39%) patients and probable sporotrichosis in 132 (61%). Incidence increased from 0.3 cases/100,000 outpatient visit-years in 2011 to 21.4 cases/100,000 outpatient visit-years in 2021; of the 216 cases, 58% (n = 126) were diagnosed during 2019–2021. The main clinical form of sporotrichosis was lymphocutaneous (63%), followed by localized cutaneous (24%), ocular (10%), multisite infections (3%), and cutaneous disseminated (<0.5%). Since the first report of CTS in Curitiba in 2011, sporotrichosis has increased substantially, indicating continuous disease transmission. Clinician and public awareness of CTS and efforts to prevent transmission are needed.

Sporotrichosis, the most prevalent implantation mycosis worldwide, is caused by fungi of genus *Sporothrix* (*1**–**4*). In some regions of Brazil, sporotrichosis has been referred to as cat disease because of its zoonotic transmission from felines. Since 1990, a new *Sporothrix* species, *S. brasiliensis*, has emerged rapidly as an agent of cat-transmitted sporotrichosis (CTS) (*5*). Initially identified primarily in Rio de Janeiro, highly virulent *S. brasiliensis* causes a notable level of epizootic disease involving cats, dogs, and humans (*5**–**12*) that is emerging and expanding geographically across Brazil (*7**–**9**,**11*) and is now a major public health problem (*12*). Originally, CTS was reported primarily in the South and Southeast regions of Brazil, but by 2022, CTS was reported in 25 of its 26 states, as well as in neighboring Argentina, Chile, and Paraguay (Figure 1) (*2*,*9*,*13*–*18*). In 2022, a case of cutaneous CTS caused by *S. brasiliensis* was reported in a veterinarian in the United Kingdom who was infected by an imported cat with sporotrichosis (*19*,*20*). 

**Figure 1:**
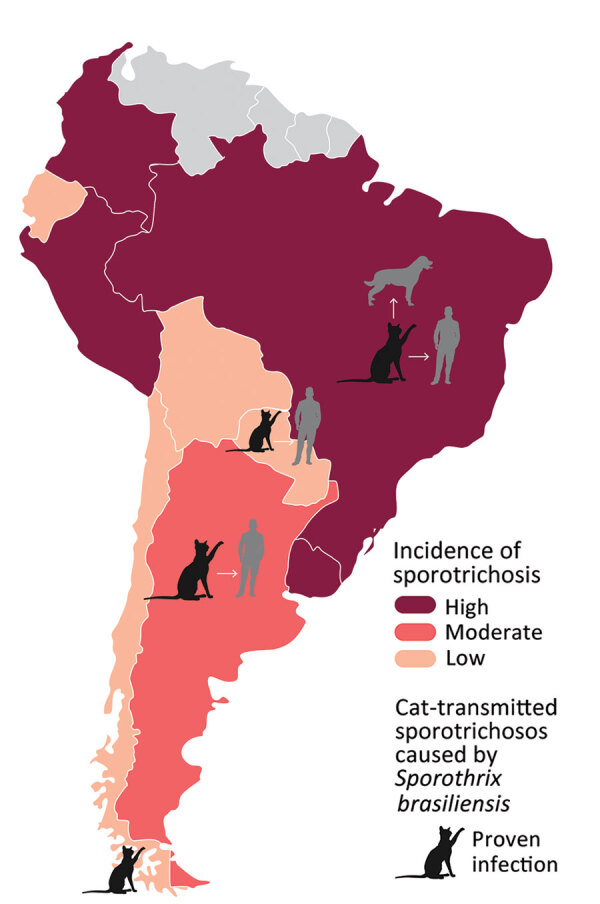
Burden of sporotrichosis in South America and distribution of cat-transmitted sporotrichosis in humans caused by *Sporothrix brasiliensis*, 2022. Cat icons indicate countries where cases of cat-transmitted sporotrichosis caused by *S. brasiliensis* have been reported; arrows indicate transmission from cats, humans, and dogs.

Rio de Janeiro state, in the Southeast region of Brazil, has the highest prevalence of CTS, >8,900 human cases reported since the beginning of the outbreak, followed by Rio Grande do Sul (South region) with 181 human cases (*14*,*15*,*21*). In Paraná state, also in the South region, public health officials and clinicians have been alarmed by the emergence of CTS, but epidemiologic and clinical data on this disease in this jurisdiction are lacking because reporting is not mandatory (*11*,*22*). Therefore, we performed a retrospective, descriptive study of human CTS to describe the characteristics of patients with sporotrichosis, based on a decade of experience in a single medical institution, the Hospital de Clínicas of the Federal University of Paraná (HC/UFPR), a tertiary referral hospital in Curitiba, Paraná’s largest city. Our study was approved by the HC/UFPR ethical committee (registration no. 12379819.4.0000.0096). 

## Methods

### Study Design 

We identified cases from the hospital database by using code B42 from the International Classification of Diseases, 10th Revision. Our study analyzed data from all proven and probable CTS cases from HC/UFPR during January 2011–May 2022. 

### Case Definition

We used case definitions approved by the Brazilian Ministry of Health (*23**–**25*). Proven human CTS was defined as microbiologic evidence (positive culture or histopathology) of sporotrichosis, presence of lesions or other symptoms compatible with sporotrichosis, and evidence of CTS transmission, including cat scratches or bites, contact with feline exudates, or exposure to sneezing by cats (Table 1) (*23**–**26*). Probable human CTS was defined as presence of compatible clinical manifestations after traumatic injury from or close contact with infected cats but lacking microbiologic evidence of sporotrichosis (Table 1) (*23**–**25*). We excluded patients with possible CTS or among whom CTS was ruled out. 

**Table 1:** Cat-transmitted sporotrichosis case definitions used in study of human cases in Curitiba, Brazil, 2011–2022*

Definition	Epidemiology	Clinical	Laboratory
Proven	History of trauma or contact with a cat with sporotrichosis	Lesions compatible with sporotrichosis	Positive culture and/or histopathology (microbiological evidence)
Probable	History of trauma or contact with a cat with sporotrichosis	Lesions compatible with sporotrichosis	Human: Negative culture and/or histopathology.† Sick cat: A) sporotrichosis diagnosed by culture and/or histopathology in a veterinarian laboratory; B) cat resident in a region with confirmed presence of cats with sporotrichosis (data verified by public health authorities)
Possible	History of trauma or contact with a cat with sporotrichosis	Lesions compatible with sporotrichosis	Absent
Non-CTS	History of trauma or contact with a cat with sporotrichosis	Lesions compatible with sporotrichosis	Negative culture and/or histopathology for *Sporothrix* spp.† Definition of other case of disease (infectious or noninfectious)

*Adapted from Guide to Health Surveillance, 5th ed (*23*–*25*). CTS, cat-transmitted sporotrichosis. †Negative culture for *Sporothrix* spp. alone does not rule out diagnosis (limitation of the culture).

### *Sporothrix* Isolate Molecular Typing

We molecularly identified at the species level all *Sporothrix* isolates obtained from patients and extracted DNA from isolates using MasterPure Yeast DNA Purification Kit (Thermo Fisher Scientific). We amplified the calmodulin (*CAL*) locus region using the degenerate primers CL1 (5′-GAR TWC AAG GAG GCC TTC TC-3′) and CL2A (5′-TTT TTG CAT CAT GAG TTG GAC-3′), then sequenced amplicons using a BigDye Terminator v3.1 Cycle Sequencing Kit (Applied Biosystems). We visually inspected sequences using the BioEdit version 7.2.5 sequence alignment editor and used MAFFT version 7 to align sequences with reference strains from the National Center for Biotechnology Information databank and MEGA version 7.0.26 software with Tamura Nei method with 1,500 bootstraps to calculate evolutionary distance. 

### Data Collection and Statistical Analysis

From hospital clinical records we collected clinical, laboratory, and epidemiologic data from cases and stored them using a standardized Microsoft Excel form. We calculated descriptive statistics to record the characteristics of the cases. We also calculated the incidence rate per 100,000 outpatient all-reason visits per year (outpatient visit-years [OPVY]). We performed analyses using Medcalc version 19.0 statistical software and used Qgis version 3.28.3 to map distribution of CTS cases by residence coordinates (latitude and longitude). We obtained graphic sources of the vector layer from the Instituto de Pesquisa e Planejamento Urbano de Curitiba, Instituto Ambiental do Paraná, and Coordenação da Região Metropolitana de Curitiba. We created kernel density maps to show hotspot areas for sporotrichosis in Curitiba; for this analysis, we established a search radius of 750 m. 

## Results

**Figure 2:**
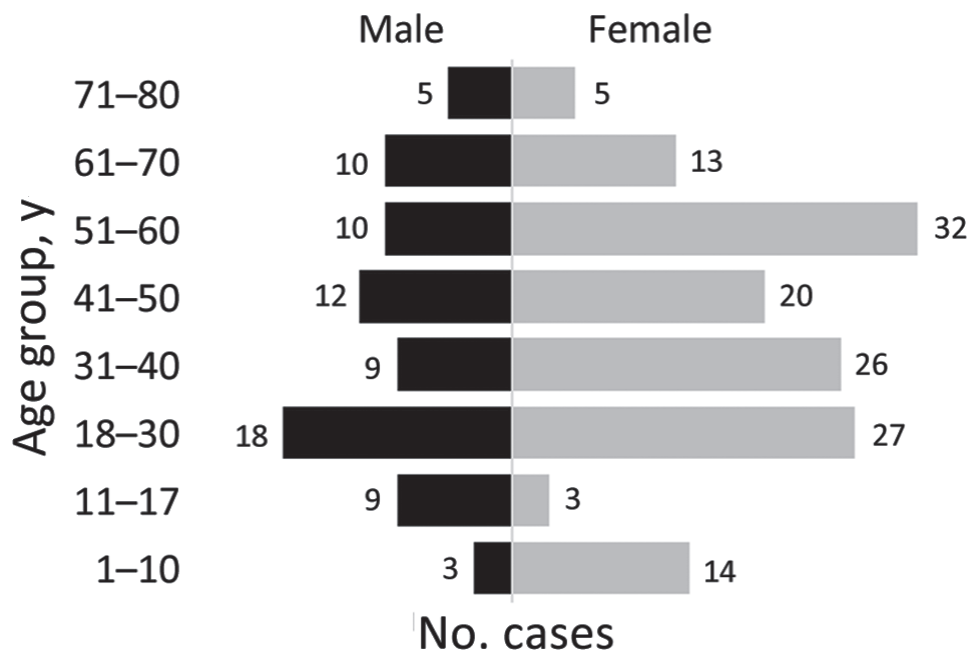
Age–sex pyramid showing the distribution of cat-transmitted sporotrichosis cases in patients treated at Hospital de Clínicas, Federal University of Paraná, Curitiba, Brazil, 2011–May 2022.

**Figure 3:**
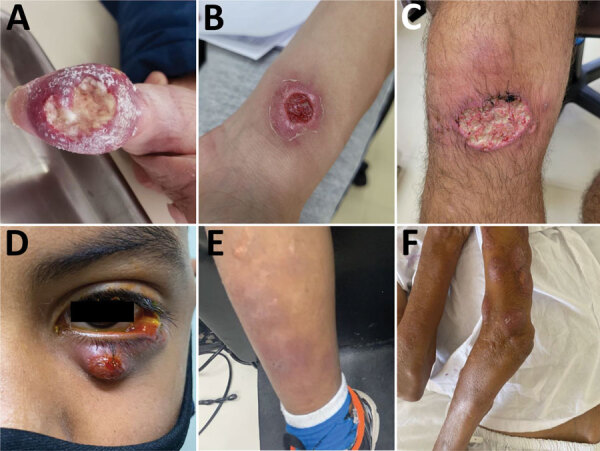
Unusual clinical manifestations of cat-transmitted sporotrichosis in patients treated at Hospital de Clínicas, Federal University of Paraná, Curitiba, Brazil, 2011–May 2022. A) Fixed cutaneous manifestation in the thumb with osteoarticular involvement. B) Fixed cutaneous manifestation in forearm with ulcer similar to primary cutaneous leishmaniasis. C) Lymphocutaneous manifestation in the knee with a large ulceration, mimicking cutaneous leishmaniasis. D) Ocular form with Parinaud ocular-glandular syndrome and dacryocystitis. E) Erythema nodosum resulting from immunoreactive sporotrichosis manifestation in the leg (same patient from panel D). F) Cutaneous disseminated manifestation in immunocompromised patient.

We included 216 CTS cases in this analysis. Case-patients were more frequently female than male (ratio 1.8:1) except in the 11–17 year age group (Figure 2). Median age among CTS case-patients was 40 years (interquartile range 22.5–56.0 years) (Table 2); 29 (13%) patients were <18 years of age. Overall, 11% of patients had occupations with disease-related risk factors, including 9 veterinarians, 5 veterinary students, 3 pet sitters, and 2 gardeners. The most frequent clinical form of sporotrichosis was lymphocutaneous (n = 136, 63%), followed by fixed cutaneous (n = 53, 25%), ocular (n = 21, 10%), mixed forms (n = 5, 2%), and cutaneous disseminated (n = 1, <0.5%). We observed that some CTS case-patients had unusual clinical manifestations (Figure 3). 

**Table 2:** Sociodemographic characteristics in study of human sporotrichosis cases in Curitiba, Brazil, 2011–2022

Characteristic	No. (%) patients
Sex	
F	140 (65)
M	76 (35)
Age range, y	
≤10	17 (8)
11–17	12 (6)
18–30	45 (21)
31–60	109 (50)
>60	33 (15)
Occupation	
Unemployed	42 (19)
Student	42 (19)
Retired	23 (11)
Domestic worker	17 (8)
Veterinarian or veterinary student	17 (8)
Administrator	11 (5)
Teacher	3 (1)
Pet sitter	3 (1)
Gardener	2 (1)
Butcher	2 (1)
Others	54 (26)

**Figure 4:**
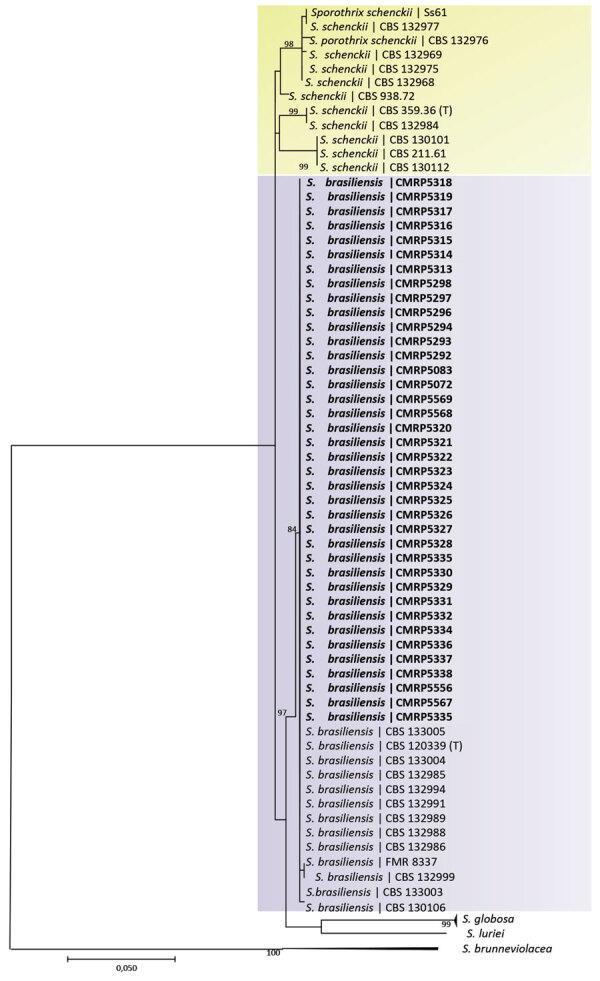
Phylogenetic analysis of *Sporothrix* spp. isolates from cat-transmitted sporotrichosis patients treated at Hospital de Clínicas, Federal University of Paraná, Curitiba, Brazil, 2011–May 2022. Tree shows analysis of clades based on calmodulin near-complete genes constructed with maximum-likelihood implemented in MEGA 7.0.26. Bootstrap values >80 from 1,500 resampled datasets are shown. Bold indicates strains isolated in this study; yellow shading represents *S. schenckii* isolates and *S. brasiliensis* isolates. GenBank accession numbers are shown in Appendix Table 1. Scale bar represents number of substitutions per site.

Overall, 84 (39%) patients had proven CTS and 132 (61%) had probable CTS. Among probable CTS cases, we tested 18 patients by microscopy and culture, and all tested negative. Laboratory testing was not requested for the 14 remaining probable CTS case-patients; the main causes for not testing were specimen unavailability for microbiology testing and previous initiation of antifungal treatment. Among the proven CTS cases, *Sporothrix* isolates were collected from direct swabbing of lesion secretions for 50 patients, skin biopsy for 29, and aspirate from abscesses for 5. Average times for positive *Sporothrix* culture results varied by specimen type: specimens collected using the swab method averaged 6 days (range 3–17 days), compared with 13 days (range 4–30 days) for specimens collected by biopsy (p<0.0001). On the basis of phylogenetic analysis of calmodulin sequence genes of 38 *Sporothrix* isolates from patients, we identified *S. brasiliensis* as the only etiologic agent (38/38 cases) of CTS during the study period (Figure 4). 

**Figure 5:**
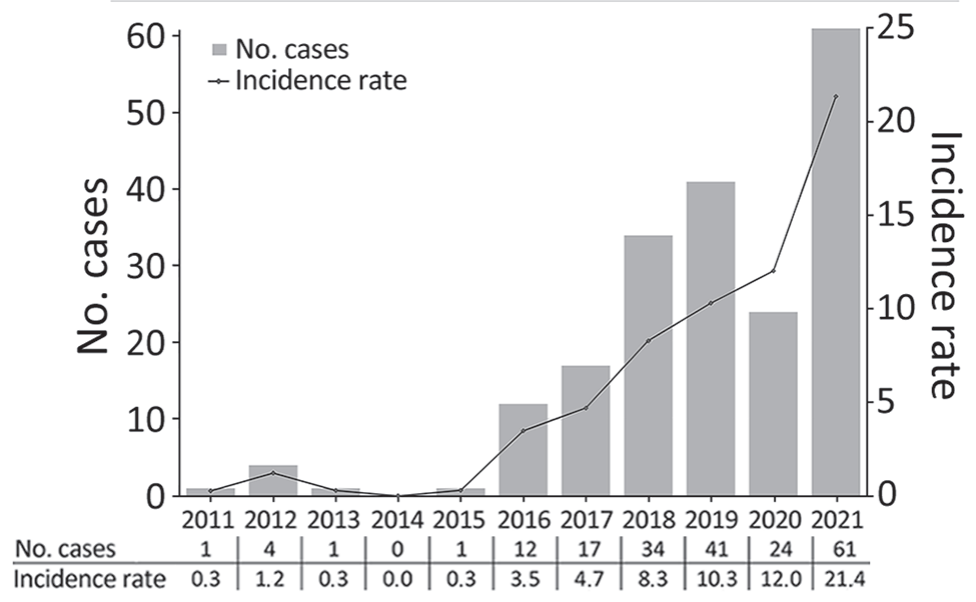
Epidemiologic curve and incidence rate (cases/100,000 outpatient visit-years) of cat-transmitted sporotrichosis patients treated at Hospital de Clínicas, Federal University of Paraná, Curitiba, Brazil, 2011–2021.

In 98% (212) of the 216 total CTS cases, infection was associated with zoonotic transmission; in those cases, the patient had direct or indirect contact with a cat with diagnosed sporotrichosis. In 4 (2%) sporadic cases, occurring in 2016, 2017, 2019, and 2021, infection was associated with saprozoic exposure with traumatic implantation from an environmental source. The first observed substantial increase in cases was from 1 in 2015 (0.3 cases/100,000 OPVY) to 12 in 2016 (3.5 cases/100,000 OPVY) (Figure 5), and incidence rates continued to increase thereafter. There were 34 cases (8.3 cases/100,000 OPVY) in 2018, almost 3 times as many as in 2016, and 61 cases (21.4 cases/100,000 OPVY) in 2021 (Figure 5). During January–May 2022, we identified 20 cases from 142,873 outpatient visits (14 cases/100,000 OPVY) (Figure 5). 

**Figure 6:**
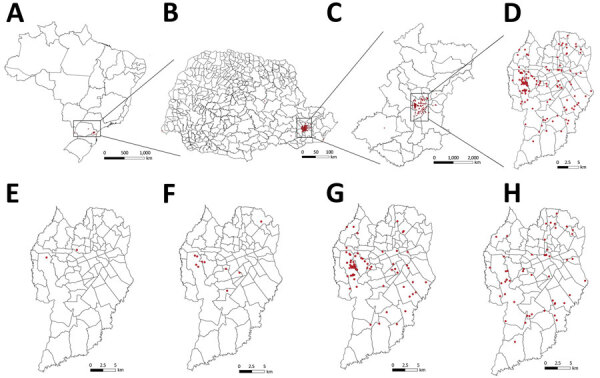
Locations of human cat-transmitted sporotrichosis cases (red dots) treated at Hospital de Clínicas, Federal University of Paraná, Curitiba, Brazil, during 2011–May 2022, and evolution of spatial distribution of cases in the city of Curitiba. A–D) Locations of all human cases in Brazil (A), Paraná state (B), Metropolitan Region of Curitiba (C), and Curitiba (D). E–H) Distribution of new human cases in Curitiba during 2011–2013 (E), 2014–2016 (F), 2017–2019 (G), and 2020–May 2022 (H).

CTS cases were reported in 15 of the 399 municipalities in the state of Paraná (Figure 6, panels A, B) and in 11 of the 29 municipalities comprising the metropolitan region of Curitiba (Figure 6, panel C). Among the 216 CTS cases identified in the state of Paraná, the metropolitan region of Curitiba accounted for 205 (95%), the city of Curitiba alone for 170 (79%) (Figure 6, panel D). We also tracked triennial distribution of cases in the city of Curitiba during 2011–2013, 2014–2016, 2017–2019, and 2020–May 2022. The first case of CTS in Paraná was identified in 2011 in Campina Grande do Sul, a municipality located in the metropolitan region of Curitiba. The first case of CTS in the city of Curitiba was identified in 2012 in the Mercês neighborhood, located in the north-central part of the city (Figure 6, panel E). Since 2016, cases have spread across the city of Curitiba and been reported in 41 of 75 districts; most cases have been identified in the northwest region of the city, but during 2020–May 2022, incidence spread throughout the city (Figure 6, panels F, G, H). We also observed clusters of CTS cases involving members of the same household. Since 2011, a total of 10 clusters involving 21 cases were identified; 7/10 CTS clusters (15 cases) occurred after the COVID-19 pandemic began in 2020. 

**Figure 7:**
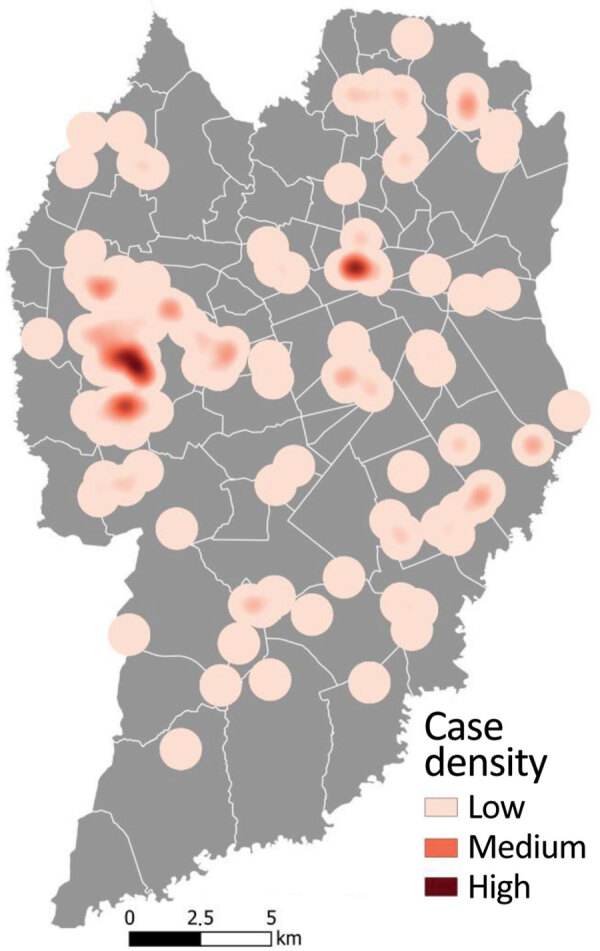
Heatmap of human cat-transmitted sporotrichosis cases using kernel density estimation in Curitiba, Brazil, 2011–May 2022. Kernel density map was created using a search radius of 750 m.

Kernel density maps identified hotspots for sporotrichosis in the city of Curitiba (Figure 7). The most critical hotspots were identified in the western part, mainly the Cidade Industrial de Curitiba (CIC) neighborhood, and north-central (city center) areas of Curitiba. CIC has an area of 44,588 km^2^ and a population of 172,909 inhabitants (density 3.9 habitants/km^2^); it is largely residential, and most residents live in houses. CIC is economically characterized as a lower middle–income area, with an average daily income of USD $4.7 (*27*). The second hotspot, in downtown Curitiba, is a combination of commercial and residential areas, composed mostly of apartment buildings. Curitiba city center comprises 3,310 km^2^ and is the most densely populated (37,234 inhabitants; density 11.2 inhabitants/km^2^) part of the city. Average daily income among residents is USD $20 (*27*). The kernel density maps of Curitiba show additional medium-density hotspots for CTS throughout other parts of the city, especially in the south, southeast, and northeast parts. 

## Discussion

During the 10-year study period, sporotrichosis incidence increased substantially, suggesting uncontrolled spread in the city of Curitiba. Brazil is facing the largest reported current zoonotic outbreak of CTS globally (*9*,*11*,*13*,*28*). CTS is a public health issue exacerbated by insufficient public health activities, such as case surveillance, animal control, and diagnostic capacity, and by lack of disease awareness among health professionals and the general population (*9*,*13*,*25*,*29*). Our study adds relevant epidemiologic information to promote better understanding of CTS in the state of Paraná, Brazil. 

We found a higher prevalence of CTS among female patients, especially in adult women involved in domestic activities. This finding is similar to those of other previous reports and is likely related to close contact with infected domestic cats (*30**–**33*). In this study, we observed an increased proportion of pediatric CTS cases; 29 cases (13%) occurred in patients <18 years of age, likely reflecting the close nature of interactions between children and domestic pets, especially cats (*23*). In addition, we found that >10% of cases occurred in persons working in professional animal care disciplines (*2*,*11*,*22*), who are at increased risk for CTS because of close contact with and exposure to contaminated body fluids from infected animals. Those professionals would benefit from increased awareness that infection in humans can occur through direct contact with body fluids from cats contaminated with *S. brasiliensis* yeast (*26*). 

Lymphocutaneous and fixed cutaneous forms were the most frequently identified clinical manifestations of sporotrichosis, consistent with findings in previous literature (*1*,*22*). However, since the sporotrichosis epidemic in Brazil began, reports of unusual clinical manifestations have been increasing (*1*,*6*,*34*). It should be highlighted that cutaneous disseminated sporotrichosis is a rare manifestation that mostly occurs in immunocompromised persons; it differs from cutaneous manifestations caused by multiple traumatic implantations, as also happens in CTS cases (*35**–**37*). We also found a relatively high frequency of ocular sporotrichosis, which occurred in 10% of cases. Ocular sporotrichosis occurs mainly because of direct contact of the ocular mucosa of a person with secretions from an infected cat, usually because of cutaneous exudates or respiratory droplets expelled during cat sneezing (*5*,*12**,**26*). Ocular sporotrichosis is characterized by acute or chronic conjunctivitis, including Parinaud ocular syndrome and dacryocystitis (*2*,*12*,*38**–**41*). We also observed that unusual clinical manifestations of CTS increased from 2018 onward, coinciding with the epidemic increase in the number of cases of the disease. On the basis of those findings, the current guidelines in Brazil recommend use of personal protective equipment, including glasses or a face shield, during contact with sick cats (*24*). 

Proven cases of CTS in this study made up slightly more than one third (39%) of all cases, compared with 65.3% in other case-series studies of sporotrichosis (*22*,*23*). This difference may have resulted from using a case definition not based solely on culture and microscopy results, which are suboptimal assays for detecting sporotrichosis in humans. Cultures from pus or secretion from lesions could be a feasible alternative; that type of test shows good performance and generates results in half the time compared with testing specimens obtained by biopsy. In addition, that type of sample is noninvasive, simple, and inexpensive. HC/UFPR has a medical institute with extensive experience in the diagnosis of sporotrichosis, and its medical professionals consequently have a high index of suspicion for this disease, increasing the chances of early detection of cases in which culture and microscopy are generally negative. On the basis of this experience, we highlight the importance of the probable case classification, because recognizing probable CTS enables rapid initiation of treatment, resulting in a positive effect on the quality of life of patient with sporotrichosis (*23*,*25*).

Molecular typing identified a single species, *S. brasiliensis*, in all proven cases in our study. The isolates were related to the species that caused the CTS outbreak in the state of Rio de Janeiro in the early 2000s and is primarily causing outbreaks throughout Brazil (*5*,*11*,*13*,*17*,*42**–**44*). Those isolates were identified by phylogenetic analysis of the near-complete calmodulin gene sequencing, showing a high degree of similarity with previously sequenced isolates from Rio de Janeiro. Additional analyses, such as microsatellite typing, multilocus sequence typing, and amplified fragment-length polymorphic fingerprinting, can improve understanding of the expansion of cases in Paraná and the city of Curitiba (*13*,*45*). Moreover, analysis of whole-genome sequencing results is a tool relevant for elucidating central concerns about the differences in infectious potential between closely related species. Comparative genomic analysis of data on tissue invasion and transmission of pathogenic sibling species has been applied to highlight genes involved in fungal adaptation to an animal-associated lifestyle (*11*,*46*,*47*)*.*

We observed an exponential increase in the incidence rate of sporotrichosis, from 0.3 OPVY in 2011 to 21.4 OPVY in 2021, a trend similar to that for data reported recently in a review of the burden of sporotrichosis in Brazil (*9*,*15*,*22*,*29*,*30*,*48*). In Brazil, the largest number of CTS cases is concentrated in the southwest and south of Brazil, including the states of Rio de Janeiro, Rio Grande do Sul, São Paulo, Espírito Santo, and Minas Gerais. However, cases of CTS have also been described in the Midwest, Federal District, and Northeast regions and in Pernambuco and Rio Grande do Norte states, clearly showing the spread of the disease throughout Brazil (*22*,*32*,*49*). 

Most cases in this study occurred in the city of Curitiba. The first case identified at HC/UFPR was in the center-north part of the city in 2012, but the first substantial increase in the number of cases, a trend that continued, was observed in 2016. After the World Health Organization declared COVID-19 a pandemic in March 2020 and lockdowns started, we observed a 37% increase in cases and also an increase in CTS clusters. The increase may have been related to higher rates of pet adoption aimed at reducing anxiety and depression because of the lockdowns (*23*). We identified 2 areas of the city of Curitiba as CTS hotspots, one area characterized by high population density and the other by social inequalities. In addition, the kernel density estimate showed low or medium CTS expanded practically throughout the city, especially during 2020–2022; areas of medium density were located in the south, southeast, and northeast parts of the city. 

The main limitation of our study was that data came from a single center; however, the data covered a decade of experience, and our findings add information based on actual case numbers rather than estimates. Since March 7, 2022, the State Department of Health of Paraná, has provided guidelines (resolution no. 093/2022) for mandatory notification of human and animal sporotrichosis cases in the state. 

Because of the zoonotic transmission of this disease, a One Health approach is necessary to control the public health effects of CTS. Integrating human, animal, and environmental health approaches through coordinated actions among microbiologists, veterinarians, physicians, epidemiologists, and surveillance authorities could benefit control efforts. Potential actions include owners restricting cats from going outside homes and having contact with street cats, providing no-cost neutering of cats in regions where *S. brasiliensis* is endemic to prevent expansion of feral cat populations, widely establishing compulsory notification of CTS cases, increasing awareness of the disease among clinicians and the public, providing easy access to treatment in human and animal cases, and cremating cat remains (*12*,*36*,*50*). Additional interventions to control CTS outbreaks could include providing access to rapid, accurate diagnostic assays and antifungal drugs, developing vaccines specifically for cats, and evaluating novel treatment strategies, particularly for treating feline sporotrichosis. Enacting those and other prevention and control measures in a timely manner is urgently needed to repress the increased incidence and spread of this serious health condition. 

Appendix Additional information from study of patients with cat-transmitted sporotrichosis Curitiba, Brazil.
