# Supplementary material for: Rising Incidence of Sporothrix brasiliensis Infections, Curitiba, Brazil, 2011–2022
Source: Emerg Infect Dis. 2023 Jul;29(7):1330–9. doi: 10.3201/eid2907.230155 (PMC10310366; doi:10.3201/eid2907.230155)
Supplement: Appendix — Additional information from study of patients with cat-transmitted sporotrichosis Curitiba, Brazil. [file 23-0155-Techapp-s1.pdf]

Article DOI: https://doi.org/10.3201/eid2907.230155

EID cannot ensure accessibility for supplementary materials supplied by authors. Readers who have difficulty accessing supplementary content should contact the authors for assistance.

Rising Incidence of *Sporothrix brasiliensis* Infections, Curitiba, Brazil, 2011–2022

Appendix

Appendix Table. Additional information about the reference strains and isolates analyzed from *Sporothrix* pathogenic clade and strains isolated from patients on Curitiba, PR.

Species	Strain reference	Geography	Source	GenBank accession CAL	Reference
S. brasiliensis	CBS 120339 (T)	Brazil	Human sporotrichosis	AM116899	(1)
S. brasiliensis	CBS 132985	Brazil	Feline sporotrichosis	KC693830	(2)
S. brasiliensis	CBS 132989	Brazil	Feline sporotrichosis	KC693846	(2)
S. brasiliensis	CBS 132999	Brazil	Feline sporotrichosis	KC693871	(2)
S. brasiliensis	CBS 133003	Brazil	Feline sporotrichosis	KC693875	(2)
S. brasiliensis	CBS 132994	Brazil	Canine sporotrichosis	KC693864	(2)
S. brasiliensis	CBS 133005	Brazil	Feline sporotrichosis	KC693878	(2)
S. brasiliensis	CBS 133004	Brazil	Canine sporotrichosis	KC693876	(2)
S. brasiliensis	CBS 132988	Brazil	Human sporotrichosis	KC693840	(2)
S. brasiliensis	CBS 130106	Brazil	Human sporotrichosis	KP101422	(3)
S. brasiliensis	CBS 132991	Brazil	Human sporotrichosis	KP101428	(3)
S. brasiliensis	FMR 8337	Brazil	Soil	AM116876	(1)
S. brasiliensis	CBS 132986	Brazil	Human sporotrichosis	KC693831	(2)
S. brasiliensis	CMRP5313	Brazil	Human sporotrichosis	ON397974	This study
S. brasiliensis	CMRP5314	Brazil	Human sporotrichosis	ON397975	This study
S. brasiliensis	CMRP5072	Brazil	Pediatric sporotrichosis	ON397976	This study
S. brasiliensis	CMRP5315	Brazil	Human sporotrichosis	ON397977	This study
S. brasiliensis	CMRP5316	Brazil	Human sporotrichosis	ON397978	This study
S. brasiliensis	CMRP5317	PR, Brazil	Human sporotrichosis	ON397979	This study
S. brasiliensis	CMRP5292	PR, Brazil	Human sporotrichosis	ON397980	This study
S. brasiliensis	CMRP5318	PR, Brazil	Human sporotrichosis	ON397982	This study
S. brasiliensis	CMRP5083	PR, Brazil	Human sporotrichosis	ON397983	This study
S. brasiliensis	CMRP5319	PR, Brazil	Human sporotrichosis	ON397984	This study
S. brasiliensis	CMRP5320	PR, Brazil	Human sporotrichosis	ON397985	This study
S. brasiliensis	CMRP5293	PR, Brazil	Pediatric sporotrichosis	OM273723	(4)
S. brasiliensis	CMRP5294	PR, Brazil	Pediatric sporotrichosis	OM273724	(4)
S. brasiliensis	CMRP5321	PR, Brazil	Human sporotrichosis	ON397986	This study
S. brasiliensis	CMRP5322	PR, Brazil	Human sporotrichosis	ON397987	This study
S. brasiliensis	CMRP5323	PR, Brazil	Human sporotrichosis	ON397988	This study
S. brasiliensis	CMRP5324	PR, Brazil	Human sporotrichosis	ON397989	This study
S. brasiliensis	CMRP5325	PR, Brazil	Human sporotrichosis	ON397990	This study
S. brasiliensis	CMRP5296	PR, Brazil	Human sporotrichosis	ON397991	This study
S. brasiliensis	CMRP5297	PR, Brazil	Pediatric sporotrichosis	OM273725	(4)
S. brasiliensis	CMRP5326	PR, Brazil	Human sporotrichosis	ON397992	This study
S. brasiliensis	CMRP5327	PR, Brazil	Human sporotrichosis	ON397993	This study
S. brasiliensis	CMRP5328	PR, Brazil	Human sporotrichosis	ON397994	This study
S. brasiliensis	CMRP5329	PR, Brazil	Human sporotrichosis	ON397995	This study
S. brasiliensis	CMRP5330	PR, Brazil	Human sporotrichosis	ON397996	This study
S. brasiliensis	CMRP5331	PR, Brazil	Human sporotrichosis	ON397997	This study
S. brasiliensis	CMRP5332	PR, Brazil	Human sporotrichosis	ON397998	This study
S. brasiliensis	CMRP5333	PR, Brazil	Human sporotrichosis	ON397999	This study
S. brasiliensis	CMRP5334	PR, Brazil	Human sporotrichosis	ON398000	This study
S. brasiliensis	CMRP5556	PR, Brazil	Human sporotrichosis	ON398001	This study
S. brasiliensis	CMRP5298	PR, Brazil	Pediatric sporotrichosis	OM273727	(4)
S. brasiliensis	CMRP5335	PR, Brazil	Human sporotrichosis	ON398002	This study
S. brasiliensis	CMRP5336	PR, Brazil	Pediatric sporotrichosis	OM273726	(4)

Species	Strain reference	Geography	Source	GenBank accession CAL	Reference
S. brasiliensis	CMRP5337	PR, Brazil	Human sporotrichosis	ON398003	This study
S. brasiliensis	CMRP5338	PR, Brazil	Human sporotrichosis	ON398004	This study
S. brasiliensis	CMRP5567	PR, Brazil	Human sporotrichosis	ON931613	This study
S. brasiliensis	CMRP5568	PR, Brazil	Human sporotrichosis	ON931614	This study
S. brasiliensis	CMRP5569	PR, Brazil	Human sporotrichosis	ON931615	This study
S. brunneoviolacea	CBS 793.73	Germany	Soil	KP017106	(3)
S. brunneoviolacea	CBS 101570	USA	Endophyte	KP017101	(3)
S. globosa	CBS 120340 (T)	Spain	Human sporotrichosis	KP101459	(1)
S. globosa	CBS 132923	Brazil	Human sporotrichosis	JF811337	(2)
S. globosa	CBS 132924	Brazil	Human sporotrichosis	JF811338	(2)
S. globosa	CBS 130104	Spain	Human sporotrichosis	AM116905	(1)
S. globosa	CBS 132922	Brazil	Human sporotrichosis	JF811336	(2)
S. globosa	CBS 132925	Brazil	Human sporotrichosis	KP101475	(3)
S. globosa	CBS 130116	Spain	Human sporotrichosis	AM116903	(1)
S. globosa	CBS 140741	China	Human sporotrichosis	KY387689	(5)
S. globosa	CBS 140743	China	Human sporotrichosis	KY387691	(5)
S. globosa	KMU 4208	China	Plant	AM399002	(6)
S. luriei	CBS 937.72 (T)	South Africa	Human sporotrichosis	AM747302	(6)
S. schenckii	CBS 359.36 (T)	USA	Human sporotrichosis	AM117437	(1)
S. schenckii	CBS 938.72	France	Human sporotrichosis	AM490340	(6)
S. schenckii	CBS 132968	Brazil	Human sporotrichosis	JX077123	(2)
S. schenckii	CBS 132969	Brazil	Human sporotrichosis	JX077125	(2)
S. schenckii	CBS 130101	Peru	Human sporotrichosis	KP101390	(3)
S. schenckii	CBS 130112	Peru	Human sporotrichosis	KP101391	(3)
S. schenckii	CBS 211.61	South Africa	Not informed	KP101393	(3)
S. schenckii	CBS 132975	Brazil	Human sporotrichosis	JQ041905	(7)
S. schenckii	CBS 132976	Japan	Human sporotrichosis	KF574464	(8)
S. schenckii	CBS 132977	Mexico	Plant	KF574467	(8)
S. schenckii	CBS 132984	USA	Human sporotrichosis	KF574470	(8)
S. schenckii	Ss61	Brazil	Soil	KF561244	(8)

References

<jrn>1. Marimon R, Gené J, Cano J, Trilles L, Dos Santos Lazéra M, Guarro J. Molecular phylogeny of *Sporothrix schenckii*. J Clin Microbiol. 2006;44:3251–6. PubMed https://doi.org/10.1128/JCM.00081-06</jrn>

<jrn>2. Rodrigues AM, de Melo Teixeira M, de Hoog GS, Schubach TM, Pereira SA, Fernandes GF, et al. Phylogenetic analysis reveals a high prevalence of *Sporothrix brasiliensis* in feline sporotrichosis outbreaks. PLoS Negl Trop Dis. 2013;7:e2281. PubMed https://doi.org/10.1371/journal.pntd.0002281</jrn>

<jrn>3. Zhang Y, Hagen F, Stielow B, Rodrigues AM, Samerpitak K, Zhou X, et al. Phylogeography and evolutionary patterns in *Sporothrix* spanning more than 14 000 human and animal case reports. Persoonia. 2015;35:1–20. PubMed https://doi.org/10.3767/003158515X687416</jrn>

<jrn>4. Queiroz-Telles F, Bonifaz A, Cognialli R, Lustosa BPR, Vicente VA, Ramírez-Marín HA. Sporotrichosis in children: case series and narrative review. Curr Fungal Infect Rep. 2022;16:33–46. PubMed https://doi.org/10.1007/s12281-022-00429-x</jrn>

<jrn>5. Moussa TAA, Kadasa NMS, Al Zahrani HS, Ahmed SA, Feng P, Gerrits van den Ende AHG, et al. Origin and distribution of *Sporothrix globosa* causing sapronoses in Asia. J Med Microbiol. 2017;66:560–9. PubMed https://doi.org/10.1099/jmm.0.000451</jrn>

<jrn>6. Marimon R, Cano J, Gené J, Sutton DA, Kawasaki M, Guarro J. *S. brasiliensis, S. globosa*, and *S. mexicana*, three new *S.* species of clinical interest. J Clin Microbiol. 2007;45:3198–206. PubMed https://doi.org/10.1128/JCM.00808-07</jrn>

<jrn>7. Fernandes GF, dos Santos PO, Rodrigues AM, Sasaki AA, Burger E, Perez de Camargo Z. Characterization of virulence profile, protein secretion and immunogenicity of different *Sporothrix schenckii* sensu stricto isolates compared with *S. globosa* and *S. brasiliensis* species. Virulence. 2013;4:241–9. PubMed https://doi.org/10.4161/viru.23112</jrn>

<jrn>8. Sasaki AA, Fernandes GF, Rodrigues AM, Lima FM, Marini MM, Feitosa LDS, et al. Chromosomal polymorphism in the *Sporothrix schenckii* complex. PLoS One. 2014;9:e86819. PubMed https://doi.org/10.1371/journal.pone.0086819</jrn>
